# Prospects of functional magnetic resonance imaging as lie detector

**DOI:** 10.3389/fnhum.2013.00594

**Published:** 2013-09-24

**Authors:** Elena Rusconi, Timothy Mitchener-Nissen

**Affiliations:** ^1^Department of Security and Crime Science, University College LondonLondon, UK; ^2^Department of Neurosciences, University of ParmaParma, Italy

**Keywords:** fMRI, lie detection, evidence, scientific validity, human rights

## Abstract

Following the demise of the polygraph, supporters of assisted scientific lie detection tools have enthusiastically appropriated neuroimaging technologies “*as the savior of scientifically verifiable lie detection in the courtroom*” (Gerard, 2008: 5). These proponents believe the future impact of neuroscience “*will be inevitable, dramatic, and will fundamentally alter the way the law does business*” (Erickson, 2010: 29); however, such enthusiasm may prove premature. For in nearly every article published by independent researchers in peer reviewed journals, the respective authors acknowledge that fMRI research, processes, and technology are insufficiently developed and understood for gatekeepers to even consider introducing these neuroimaging measures into criminal courts as they stand today for the purpose of determining the veracity of statements made. Regardless of how favorable their analyses of fMRI or its future potential, they all acknowledge the presence of *issues yet to be resolved*. Even assuming a future where these issues are resolved and an appropriate fMRI lie-detection process is developed, its integration into criminal trials is not assured for the very success of such a future system may necessitate its exclusion from courtrooms on the basis of existing legal and ethical prohibitions. In this piece, aimed for a multidisciplinary readership, we seek to highlight and bring together the multitude of hurdles which would need to be successfully overcome before fMRI can (if ever) be a viable applied lie detection system. We argue that the current status of fMRI studies on lie detection meets neither basic legal nor scientific standards. We identify four general classes of hurdles (scientific, legal and ethical, operational, and social) and provide an overview on the stages and operations involved in fMRI studies, as well as the difficulties of translating these laboratory protocols into a practical criminal justice environment. It is our overall conclusion that fMRI is unlikely to constitute a viable lie detector for criminal courts.

## Introduction

In recent years researchers in cognitive neuroscience have started to investigate the neural basis of complex mental processes including moral beliefs, intentions, preferences, self-knowledge, social interactions, and consciousness. Influential neuroscientists are introducing the idea that our traditional notions of crime and punishment (and the laws built upon them) should be challenged, and if necessary modified, to make them more *human-friendly*. Recent empirical findings with neuroimaging techniques challenge the central idea of free will around which much of the criminal law has been shaped (see e.g., Gazzaniga, 2008). Additionally, structural MRI evidence is making inroads in courts around the World [see e.g., *Commonwealth of Pennsylvania v. Pirela*, 2007; *Caso Bayout—Corte d'Assise d'Appello di Trieste (n.5/2009) del 18 settembre 2009*; *Tribunale di Como (n.536/2011) del 20 maggio 2011*] and it seems that not before long functional Magnetic Resonance Imaging (fMRI) scans will be routinely requested by the defense when searching for either mitigating factors, such as anatomo-functional abnormalities, and/or the presence of any crucial memories when self-reports can be doubted (e.g., Abbott, 2001, 2007; Hughes, 2010). On similar grounds, and concomitant with attempts to promote fMRI as a mind-reading tool (see Logothetis, 2008, for a specialist overview), fMRI has been proposed as a possible state-of-the-art tool for detecting both malignancy and deception in criminal courts even though it has not yet been considered admissible evidence (e.g., *US v. Semrau*, 2010; http://blogs.law.stanford.edu/lawandbiosciences/2010/06/01/fmri-lie-detection-fails-its-first-hearing-on-reliability/, also see Sip et al., 2007, 2008 and Haynes, 2008, for contrasting specialist views on applications in lie detection). In addition to raising questions regarding fMRI's reliability as a lie detecting tool according to scientific standards, such advocacy raises ethical and legal issues that are common to any putative lie detection technology thus engaging the attention of lawyers, ethicists, and philosophers.

Despite all these concerns fMRI is already being advertised as a scientifically proven lie detector by private companies having strong links with academia (see No Lie MRI—http://noliemri.com/ and CEPHOS—http://www.cephoscorp.com/), one that has not (yet) been subjected to the same regulation as the polygraph and thus is not considered an illegal means of assessment in pre-employment settings. As “trust” is increasingly prioritized in certain business sectors, top-tier corporations may be tempted to assess the trustworthiness of their key current and future employees by requesting they undergo a lie detection test via fMRI. However, in the more conservative criminal justice sector, several hurdles confront any use of fMRI as a viable lie detector. We will attempt herein to provide a realistic and accessible evaluation of such hurdles by discussing those questions raised by fMRI use for lie-detection purposes in criminal courts.

But firstly in the following two sections we take a brief look at the basics of this technique in order to gauge an impression of what types of evidence fMRI currently may and may not be able to provide. For although most neuroscientists would agree that fMRI should not be used as a lie detector, especially within its current form (e.g., Grafton et al., 2006; Tovino, 2007), the debate has recently seen the identification of a possible route toward the use of fMRI for lie detection by separating scientific from legal standards (Schauer, 2010) or basic from translational research (Langleben and Moriarty, 2013).

## Functional magnetic resonance imaging: basics

fMRI is one of the most popular measurement techniques in cognitive neuroscience. It has been in use for about 20 years and is qualified as *correlational* because it records brain states in parallel with ongoing mental activity and/or behavior, thus permitting the establishment of correlational links between them. However, it does not allow researchers to establish a causal connection between brain states and behaviors or supposed mental processes. In most fMRI studies, brain states are the dependent variable measured during manipulation of the stimulus/task condition. Whether any specific local or systemic pattern of brain states is a necessary determinant of its associated behavior it cannot be determined with fMRI only. For this reason, fMRI is routinely used in basic research as a mainstay method to measure brain function and its data are often triangulated with data from complementary techniques (e.g., event-related potentials, transcranial magnetic stimulation), in a quest for converging evidence about mental processes and brain substrates.

As implied by its name, fMRI makes use of strong magnetic fields to create images of biological tissue. Depending on the *pulse sequence*^1^ of the electromagnetic fields it generates, an MRI scanner can detect different tissue properties and distinguish between tissue types. Scanners are used to acquire both *brain structural* information (e.g., allowing a fine distinction between white and gray matter, producing images of the static anatomy of the brain), and *functional*^2^ information such as measurements of local changes in blood oxygenation within the brain over time; the most common form of fMRI study. Because blood oxygenation levels change rapidly (i.e., after 1–2 s) following the activity of neurons in a brain region, fMRI allows researchers to localize brain activity on a second-by-second basis and within millimeters of its origin (Logothetis and Pfeuffer, 2004). These changes in blood oxygenation occur naturally and internally as part of normal brain physiology and, because the pulse sequence does not alter neuronal firing or blood flow, fMRI is considered a non-invasive technique (Huettel et al., 2009).

Central to cognitive fMRI studies are the concepts of differences and similarities between maps of *blood oxygenation level-dependent (BOLD) signal*^3^ that are recorded in concomitance with different experimental conditions. In classical fMRI designs and in most of the available lie detection studies BOLD responses are evaluated in relative terms as the result of a contrast between two or more conditions. For example, maps of the BOLD signal that are recorded while a participant is lying can be contrasted with either maps recorded when the participant is at rest or when is telling the truth. Inferences about the neural correlates of lying are typically drawn from an analysis of the pattern of differences and/or similarities between BOLD signal maps across *lying* and *not-lying* conditions^4^. In principle, any design difference (e.g., the use of a different stimulus or the requirement of additional mental operations given the same stimulus) between the lying condition and any other condition with which it is compared might lead to the recruitment of different brain regions to perform the task. Therefore, the more accurately the *not-lying* and *lying* conditions are matched, the more precise the conclusions that can be drawn about the neural correlates uniquely associated with lying. While this type of analysis is not the only possible or optimal way to draw informative inferences from fMRI data (e.g., Sartori and Umiltà, 2002) such contrast between conditions is a basic standard in fMRI research. We would like to draw the attention here on the fact that the possibility to interpret as specific correlates of lying any findings ultimately resides in the original choice and design of experimental and control conditions. More recent approaches to the discrimination between *lying* vs. *not-lying* correlates include data-driven pattern classification algorithms (e.g., Davatzikos et al., 2005; Kozel et al., 2005), which are bound to the possibility of an independent and objective classification of lie vs. truth (Sip et al., 2008). Common to all approaches within brain data analyses however is that that which is identified as the “correlates of lying” even at the individual level would be expected to emerge *across several lying trials* thereby capturing similarities across different instances of “lying,” rather than simply representing singularities associated with an *individual instance* of lying.

When evaluating fMRI evidence, with an eye on applying it to a real-world problem, it is important not only to be aware of basic experimental design principles but also of the peculiar requirements of the technique and its limitations (Spence, 2008). In this regard both scanner reliability and staff technical skills are fundamental to the internal validity^5^ of fMRI-testing protocols. This is often an issue since control conditions need to be carefully matched to experimental conditions in order to unequivocally isolate the *construct of interest*^6^. However, even with an elegant design the reliability and localization of BOLD signals depends on the extent to which participants perform their tasks accurately, consistently, and in compliance with all instructions (for example, not moving their head as movement will degrade the image). To double-check participant's compliance, behavior should be monitored and recorded during the scanning session whenever possible (e.g., by recording reaction times and accuracy in a task), and in experiments involving arousal or emotional stimuli, skin conductance, heart-rate or salivary hormones could be also monitored to provide converging information.

The outcome of data analysis is a function of a series of consensus-based decisions, including options and parameters chosen for realignment, normalization, and smoothing, statistical models for analyses, and the associated correction criteria that can be more or less conservative. Finally, strategic decisions may guide choice of the evidence that will find its way in the final report on a peer-reviewed journal. Although raw data may be requested by anyone for further analyses, most readers will exclusively rely on the information provided in a polished report. Furthermore, in very competitive scientific environments there is no incentive for investigators to try and replicate their own findings as journals typically promote the publication of novel designs rather than replications (e.g., Giner-Sorolla, 2012), and in real practice it is very unusual to see a brain imaging experiment precisely repeated within and between laboratories. This may prove especially problematic when trying to identify a well-known and reliable protocol for potential applications in the real world. Finally, numerous safety exclusion criteria apply which limits the generalizability of fMRI results and prevents its universal use (see a typical participant screening checklist at http://airto.hosted.ats.ucla.edu/BMCweb/Consent/SafetyScreen.html).

In summary, protocol design determines how fMRI evidence can be interpreted, full compliance is required from participants, and the final evidence reflects choices, assumptions and data transformations based on current scientific standards and consensus criteria but also on publication strategies. Finally, not everybody can undergo fMRI. So the question remains, can its potential contributions as a lie detector outweigh its intrinsic limitations?

## The lying brain

Many people believe they are very good at detecting deceit and that certain signs give away when somebody is lying: liars would talk too much and tell stories far more elaborate and detailed than required by the context; they would never gaze interlocutors straight in the eyes or would stare at them too intensely; or they would cross their arms or their legs; or a combination of behaviors (e.g., Houston et al., 2012). Yet studies show that the vast majority of onlookers correctly distinguish *truth* from *lies* when told by a stranger only about 54% of the time (i.e., they are only slightly better than chance). Notably, this same level of (in)accuracy holds true even for professional categories such as lawyers, policemen, magistrates, and psychiatrists (Bond and DePaulo, 2006).

Conversely, the ability to lie develops spontaneously (it is typically absent in children with neuro-developmental impairments, like autism). Lying is fundamental to healthy behavior, as shown by the disastrous social interactions of patients with orbito-frontal lesions. Indeed some of these patients become notoriously tactless—which in a final analysis can be achieved by always being completely frank and honest. The literature on orbito-frontal patients suggests in turn that the ability to lie depends on the integrity of localized neural circuits (e.g., Damasio, 1994).

Recent attempts have been made with fMRI to specify the neural correlates of lying or deception (see Christ et al., 2009; Abe, 2011 and Gamer, 2011 for recent overviews and meta-analyses; see Sip et al., 2007 for a discussion on deception and lying from a cognitive neuroscience perspective). In one of the typical experiments, researchers ask participants to answer truthfully to some questions/stimuli and lie in response to others. The BOLD contrast^7^ between the two conditions (i.e., the pattern of BOLD signals detected when the participant is lying minus the pattern of BOLD signals detected when the participant is being truthful; also indicated in the specialist literature as Lie > Truth) is expected to enable the identification of brain regions whose activation is significantly correlated with lying. Accordingly, several studies identified a network of parieto-frontal^8^ areas that are significantly more engaged when the individual is lying. As the opposite contrast (i.e., Truth > Lie) does not usually detect any regions that are significantly more engaged, most neuroscientists infer that lying requires extra-effort compared to responding truthfully. Such extra-effort is possibly aimed at inhibiting the truth and/or producing an alternative response that sounds realistic enough. In studies employing ecologically plausible stimuli, activation of regions in the limbic system (a deep brain structure traditionally associated with emotional responses) has also been associated with lying (e.g., Hakun et al., 2009). Note however that this does not imply in any mechanistic way that a person is lying when the same region of the limbic system or network of parietofrontal areas activates during a task (e.g., Poldrack, 2006, 2010; see also following *Scientific Hurdles* section, point 1). Finally, a great part of research on the neural correlates of deception has been focused on group-level results (i.e., results that are obtained by averaging data from several participants), whereas any real-world application would require a differential approach (i.e., it should provide evidence that is informative and predictive at the individual level).

Within a basic cognitive neuroscience perspective, fMRI research on deception can indeed aspire to provide correlation maps that possibly reflect the difference between deceitful and truthful responses. In order to obtain knowledge about the anatomo-functional substrates that are causally related to lying, and disambiguate potentially spurious activations, evidence would need to be collected with complementary techniques (e.g., with neurological lesion or non-invasive brain stimulation studies). fMRI is thus useful inasmuch as it hands over to techniques with complementary inferential power a map for (1) identifying cortical networks that play a necessary role in deception, and (2) testing their role by directly manipulating an individual's ability to deceive. This information could then feed back into fMRI maps and enable the identification of the most relevant correlates of lying for applicative purposes. The ability to establish causal links between brain substrates and behavior resides in the fact that the functionality of the brain tissue underlying stimulators can be temporarily modulated (e.g., see ; Nitsche et al., 2008; Sandrini et al., 2011). For example, by modulating the activity of frontal lobe areas with non-invasive brain stimulation, Priori et al. (2008) were able to interfere with intentional deception by slowing down the production of untruthful responses (see also Mameli et al., 2010). Karim et al. (2010) could enhance the ability to lie by modulating activity in a contiguous part of the frontal lobe, the anterior prefrontal cortex. It thus seems possible to manipulate efficiency in lie production by targeting specific brain regions (see Luber et al., 2009, for a discussion of related ethical implications), although careful task analysis, replication and clarification of the underlying mechanisms of action of non-invasive brain stimulation techniques need to be carried out before endorsing any mass applications. This should suggest how in basic neuroscience (1) fMRI can contribute to our models of the brain substrates of lying, however for completeness its evidence is best integrated with evidence from complementary techniques, (2) fMRI evidence alone does not provide compelling evidence as to whether certain neural substrates are strictly necessary to the process of lying. Other techniques may help restrict the focus to a subset of potential substrates.

As a final point it is worth remembering that in basic research a participant's compliance with instructions is almost taken for granted as there is no rational reason why a participant might benefit from not following them. Quite the opposite situation may arise in a criminal forensic setting however, whereby it is not difficult to imagine that either intentional (e.g., adopting countermeasures) or non-intentional (e.g., due to alterations in one's emotional state) factors may lead to inconclusive results. In this respect, a recent study by Ganis et al. (2011) has eloquently shown how easy it is to “fool” an fMRI test for participants who have been trained in the use of task-tailored countermeasures.

## fMRI as lie detector in criminal courts

### The scientific hurdles

Legal systems are not new to influences from the cognitive neurosciences. For example, admissible MRI evidence showing the absence of frontal lobe maturation in the brains of teenagers contributed to the elimination of the death penalty for minors in some US states (frontal lobes are causally implicated in decision-making and the control of impulsive reactions; e.g., Damasio, 1994; Coricelli and Rusconi, 2011). Additionally structural brain scans are widely admissible at sentencing and are now almost invariably present in capital cases. However, when it comes to lie detection not all procedures have proven acceptable with polygraphs failing to attain general admissibility in criminal courts^9^ with the exception of New Mexico.

Despite this final fact, in 2006 two private bodies *No Lie MRI* and *Cephos Corporation* were launched with the goal of bringing fMRI lie detection to the public for use in legal proceedings, employment screening, and national security investigations. Detection accuracy was claimed to be as high as 90% (compared to a purported 70% for polygraphs). Attempts are being made to admit fMRI evidence in criminal courts; for example at the end of 2009 tests performed by No Lie fMRI were presented as evidence by the defense in a child protection hearing to prove innocence claims of a parent accused of committing sexual abuse. Had they been admitted that would have been the first time fMRI was used in an American court (Simpson, 2008). They were not but it might only be a matter of time before judges form the opinion that fMRI may provide relevant scientific evidence (Aharoni et al., 2012) opening the door to their wider admissibility.

Within this and the following sections we summarize and bring together the multitude of hurdles which need to be overcome before fMRI can ever be successfully integrated into criminal trials. Our discussions are primarily restricted to the English common law system of adversarial justice as applied throughout the United Kingdom, the United States, and Australia amongst others, as opposed to the continental European mixed adversarial-inquisitorial civil law systems. This decision is based on the particular nature of adversarial trials, with its competing prosecution and defense counsels who in turn can engage the services of competing expert neuroimaging witnesses, which may exacerbate some of the issues surrounding fMRI evidence discussed herein.

Legally, for scientific evidence to be admissible in criminal trials it must meet the legal standards as set down in the relevant jurisdiction, be these common law requirements such as either the test under *Frye v United States* (1923: 293 F.1013) or the succeeding requirements under *Daubert v Merrell Dow Pharmaceuticals Inc.* (1993: 509, U.S.579) as applied in *Kumho Tire Co. v Carmichael* (1999: 526, U.S.137), the presence of statutory requirements, international conventions, Federal Rules of Evidence, or any permutation of these. Drawing from these various requirements there are general principles that scientific evidence must be both *relevant* and thereby possessing probative value, as well as being *reliable*. It is primarily this second concept of reliability that is our focus here.

Within the specific constraints of the criminal law we can comprehend scientific evidence as being reliable if, amongst other things: the methods and results are both consistent and consistently applied; the accuracy of results meets an acceptably high standard while both false positives and false negatives are minimized; what practitioners *believe* is being measured *is actually* being measured; the processes being measured are both understood by scientists and are agreed upon by scientists working in the field or who choose to examine the processes; and the scientific processes being relied upon apply equally to all individuals regardless of any internal or external traits or influences, or if there is variation this has been addressed in relation to the individual at hand. While these requirements may appear somewhat ill-defined to the objective scientist, they reflect the style of judge-made legalistic tests whereby relatively broad requirements may be set down. Within the field of law this flexibility is not seen as a vice as it both allows a future court to judge a case on its merits and does not undermine the role of the jury as the final arbiter of truth.

From the published fMRI literature it unavoidably emerges that fMRI technology has not reached this *reliability* threshold. Issues which require addressing by cognitive neuroscientists are set out below:

1. Assumptions and inferences underlying fMRI processes and technologies need to be confirmed (or dispelled) so as to give credence to the scientific claims being made. Cognitive neuroscience, for example, assumes that complex thoughts have a physical counterpart that is both accessible and interpretable with technologies such as fMRI (Erickson, 2010). Many fMRI researchers operate on the basic assumption that lying involves additional efforts than telling the truth, which in turn can be signaled by heightened blood flow in specific brain regions (Gerard, 2008). However, several fMRI studies have been employing “reverse inference” as a central feature, whereby the activation of certain brain regions (X) is taken as evidence of a particular cognitive function (Y). As thoroughly discussed by Poldrack (2006), such inferences are only deductively valid if brain state X only occurs when cognitive function Y is engaged (i.e., if a selective association between X and Y is established), yet this one-to-one matching is not the case. Rather many-to-many matching of brain states to mental states are observed, and thus valid reverse inferences cannot be made here. What is required first of all is the creation of a robust “cognitive ontology” specifying the component brain operations that comprise specific mental functions, even before trying to establish univocal associations with functional anatomy (Poldrack, 2010). Furthermore, data-driven pattern analyses approaches (e.g., Haynes, 2008), although more current in terms of the methodology and less constrained by theoretical assumptions, still rely on the objective identification of what a lie is (e.g., Sip et al., 2008). However, this is not always possible and is especially unlikely in forensic contexts where lie detectors would be employed when neither facts nor subjective intentions can be directly verified. The validity of underlying assumptions must be addressed and a wide consensus reached within the scientific community before possible applications of the technology can achieve broad credibility.
2. To achieve internal validity, it needs to be conclusively determined that what is being measured is actually evidence of deception and not unrelated cognitive processes, and this needs to be determinable for each and every response given by every future individual undergoing fMRI questioning when operational. Contrary to public expectations lie detectors like fMRI are not *mind readers*, do not actually detect deception, and will never provide details of what has actually happened in complicated cases. Rather they merely detect and measure manifestations of thoughts through changes in oxygenated blood which proponents consider denotes lying (Andrewartha, 2008; Holley, 2009). What fMRI lie detection actually depends upon is an ability to detect the suppression of competing responses, and yet remains hamstrung by the inability to determine what these competing responses are and what the suppression of these implies. As pointed out by Grafton and colleagues, “[m]any defendants [while testifying] do inhibit their natural tendency to blurt out everything they know. They are circumspect about what they say. Many of them also suppress expressions of anger and outrage at accusation. Suppressing natural tendencies is not a reliable indicator of lying, in the context of a trial” (Grafton et al., 2006, pp. 36–37). Critics have also gone on to argue that any exhibited increase in blood flow detected by fMRI may result from alternate neurological process such as anxiety, fear, or other heightened emotional states which are unrelated to the question of deception. In other words, just because the prefrontal cortex is activated during deception it does not follow that every time the prefrontal cortex activates the individual is lying (Fox, 2009; Moreno, 2009). Furthermore, assuming increased oxygenated blood flow in specific brain regions denotes deception, scientists have not agreed with a degree of precision as to what these specific regions are (Gerard, 2008). Should cognitive neuroscientists successfully address these questions, the machines and processes they develop will need to be constantly identifying and correcting for extraneous mental activity during the questioning process of criminal suspect and witnesses, otherwise any results will be an open target for legal challenge by opposing expert counsel.
3. The question of individual differences affecting fMRI results needs to be answered. The importance of this issue for a technology seeking both legitimacy and broad application cannot be underestimated. Neuroimaging devices need to be able to identify and correct for variances in individuals' brains and not operate on shared but unproven assumptions that all or most brain's process lies similarly (Ellenberg, 2009; Holley, 2009). This includes correcting for variations in brain processes based on age; particularly juveniles. They need to be able to cope with the types of individuals usually encountered by law enforcement officers, including substance addicts, those with high incentives to lie, and those with mental disorders. Doubts already exist as to whether fMRI would be usable for those presenting with conditions such as delusions and amnestic disorders with confabulation (Langleben et al., 2006). Finally there is the issue of possible differences in outputs resulting from the social diversity of those tested, given that what is considered a lie is a matter of social convention which may vary on a cultural basis (Holley, 2009).
4. The question of whether subjects or questioners can manipulate the fMRI baseline or response data needs to be addressed. To measure neurophysiological changes in the brain, neuroimaging devices must be able to create a reliable baseline against which comparisons can be formed. In this regard fMRI depends upon the cooperation of the subject and is highly vulnerable to countermeasures (e.g., Ganis et al., 2011). Trained participants can alter test results by engaging in some taxing activity like mental calculations during control sequences which will enormously reduce the power of the contrast between truthful statements and lies. Furthermore, it is not yet clear whether extensive rehearsing of a story which subsequently requires virtually no mental effort to retell will diminish detection rates (see Ganis et al., 2003 for a first attempt to distinguish spontaneous from rehearsed lies; Gerard, 2008). A separate issue of manipulation relates to both experimenter and questioner expectancy effects. Consider a scenario whereby fMRI evidence is considered admissible and a criminal suspect is being questioned while undergoing an fMRI. Undoubtedly, the suspect will be influenced by the questioner's expectancy of guilty (for example via the questioner's tone of voice, their reactions, mannerisms, etc.). Accordingly, these experimenter effects (or questioner expectancy effects) are likely to influence the suspect's neurological processes. They would need to be both recognized and treated as noise, making it more difficult to determine what the observed brain activity actually means.
5. The subjectivity inherent in fMRI analysis algorithms needs to be acknowledged and these algorithms opened up for scrutiny. It has been claimed that fMRI output is superior to previous assisted lie detection methods partly because of the automated interpretation of results with computer algorithms which reduce the risk of human error by minimizing experimenter bias and subjectivity (Bilz, 2008–2009; Gerard, 2008). However, algorithms are not purely objective artifacts; they encapsulate and reproduce all the subjectivity, bias, and assumptions of the programmers, and some of these may differ each and every time they are applied. This can introduce errors that bias results in an unevenly distributed way across individuals. Furthermore, as Simpson (2008) notes, separate research groups have devised their own independent statistical methods for identifying brain activity they each believe consistent with lying. This development of diverse approaches increases the variability of results and the general reliability of the technique (see Bennett and Miller, 2010, for a thorough discussion of the issue); the consequences of which should not be underestimated and will be amplified in an applied setting. For if competing (but accepted) algorithms produce conflicting results when interpreting fMRI questioning data then opposing prosecution and defense experts will each exploit the one which best serves their purposes in court leading to a stalemate of probative value and a negation of fMRI evidence.
6. We need to determine the percentage of the population who for various reasons are unable to undertake an fMRI, as well as the nature of those reasons. fMRI is highly sensitive to movement, requiring subjects remain virtually motionless for long periods of time during questioning for the slightest head movement can wreck the resulting image. According to a review by Alexander (2007) of fMRI trials published between 2001 and 2006, ~20% of subjects (38 of 192) were rejected because of head motion artifacts or insufficient data. Add to this the physical construction of MRI machines which involves the use of powerful magnets and the number of groups who will not be able to undergo fMRI questioning grows. This includes those with medical conditions such as Parkinson disease which prevents then remaining still without medication, those suffering claustrophobia, people with medical implants, metal pins, piercings, and shrapnel; all of which may preclude fMRI questioning (Gerard, 2008; Holley, 2009; see Appendix A).
7. Questions over the methodological validity of past and future fMRI studies must be answered. Many of the fMRI trials to date have compared group differences rather than individuals, and the few accuracy levels reported range between 78 and 90%. This discrepancy within detection rates counts against fMRI gaining admissibility within criminal trials and will not be remedied until more studies are published for peer review (Gerard, 2008). Furthermore, according to Moreno (2009) a recent meta-analysis of existing neuroscience data taken from published studies revealed that more than half of these employed defective research methods producing dubious results as a result of distorted data and biased correlation analysis; specifically *non-independence error* (see Vul et al., 2009).
8. To attain external validity, experiments need to be applicable beyond highly controlled laboratory settings to confrontational, emotional “high-stakes” criminal justice situations. Criminal investigations and trials are confrontational and may represent high personal stakes for those involved. This will affect the individuals' mental state and underlying neurological processes (see e.g., Sip et al., 2010, 2012; for evidence of modulation on both deception behavior and its brain correlates in social contexts). As Andrewartha (2008, p. 93) states, “[t]his perhaps explains why considerable judicial criticism has been made of the purported reliability of utilizing lie detector machines in litigation. The propriety of equating simulated scientific testing with real life scenarios for the purpose of evidence is highly questionable.” Unless researchers can show fMRI testing is suitably robust for the criminal law setting then this technology will remain unacceptable. Randomized controlled trials with currently available testing protocols may not be a straightforward solution to this. In addition to result interpretation problems and the current lack of a comprehensive model of deception, “translational validation” requires access to real-world situations with minimal interference and the possibility to derive an objective index of performance for deception detection. The outcome of court proceedings, for example, could not be taken as an objective parameter for the discrimination between lie and truth (whatever lie detection task is being translationally validated). It is instead already possible to predict that the introduction of fMRI evidence will significantly influence juror decision-making, if unchallenged (see e.g., McCabe et al., 2011).

According to skeptics the enthusiasm for brain imaging and related “mind reading” applications largely overestimate its current ability to identify unique neural correlates of complex mental functions such as lying (but see Haynes and Rees, 2006; Haynes, 2008). Brain activations look extremely persuasive but they result from a long series of manipulations, assumptions, and interpretations. A precise and robust model of the mental processes involved in lying should guide hypotheses about brain activations, however such a generally accepted model remains absent. In addition, lies can be of different types (i.e., denying an event that has occurred vs. making up a slightly different story vs. telling a truth which will be interpreted as a lie; for example, think of a betrayed partner asking “with whom have you had dinner last night?,” and the cheater sarcastically replying: “with my lover, obviously!” thereby telling the truth with the intent to make it sound like a lie). The context of basic fMRI experiments is artificial and one often has to sacrifice external for internal validity, and any attempts to make them more similar to real world scenarios will almost inevitably undermine internal validity. Finally the available literature cannot be generalized to all populations for lie detection protocols have not been tested on juveniles, the elderly, or individuals with problems of substance abuse, antisocial personality, mental retardation, head injury, dementia.

In summary, while fMRI may be a useful research tool in combination with other techniques to clarify the mechanisms involved in lying, and its degree of sensitivity and specificity in lie detection may be higher than that of the polygraph, most scientists currently agree that fMRI research evidence is still weak and lacks both external and construct validity (Spence, 2008). We also must conclude that the current state of the science does not at this time meet the legal standards for admissibility in court proceedings (see Simpson, 2008 and Merikangas, 2008, for exhaustive discussions).

### Legal and ethical hurdles

Since the 1920s proponents of assisted lie detection technologies have been predicting their inevitable acceptance by courts; first for polygraphs and now for neuroimaging technologies (Gerard, 2008), however to date fMRI evidence has never successfully been admitted in court for determining the veracity of statements by witnesses or defendants. This reflects the deep skepticism held by the judiciary as to the reliability of assisted lie detection techniques. This skepticism is partly borne out of the failure of the polygraph and now threatens to taint this new generation of neuroimaging technologies. The perverse irony for the cognitive neuroscientists who have been developing these new technologies in a conscious effort to address the legal short-comings of polygraphs, is that while techniques like fMRI might well-tick the boxes of reliability and objectivity when perfected, the solution of bypassing physiological responses in favor of the direct recording of neural activity may itself constitute grounds for the judiciary to reject neuroimaging technologies. Not because such solutions will necessarily lack reliability or objectivity, but because they potentially infringe other human/constitutional rights and legal principles. The developers of neuroimaging technologies need to acknowledge and engage with these legal issues *before* they seek to impose their new techniques into criminal courts if they are to maximize their chances of winning over the already skeptical judicial gate-keepers. For should they fail to find a way to square their new technologies with the existing legal principles set out below, then without legislative intervention their technologies will remain excluded from criminal courts.

1. Possible constitutional and human rights violations (illegal search, right to silence, freedom of thought, right to privacy, human dignity, right to integrity of the person, and protection of personal data):
  Looking across various common law legal systems, a number of constitutional principles, and human rights conventions^10^ will be engaged to differing degrees within different jurisdictions by the neuroimaging processes of fMRI. Ultimately without legislative intervention it will be the respective national courts who will be forced to rule on each of these issues, either when parties first seek to introduce fMRI evidence of statement veracity into criminal trials, or upon appeals to the first convictions/acquittals where this technology played a material part in arriving at a verdict. It is not our intention to examine each of these in depth here, rather to discuss broadly the various legal hurdles which must be addressed if fMRI is to find its place within criminal trials for determining the veracity of statements made.
  The first set of issues is whether fMRI questioning constitutes a *search* of the subject, and when such a search will be considered lawful or unlawful. Discussions in this area tend to center on the US Constitutional Fourth Amendment protecting against unreasonable or unlawful searches (see Pardo, 2006, and Holley, 2009, amongst others for in-depth discussions on this point). A view exists that neuroimaging techniques will constitute a legitimate search under established legal doctrine should neural activity be equated to other forms of physical evidence gathered from the human body, such as blood or DNA sampling, fingerprints, voice sampling, etc., providing probable cause exists justifying such sampling (Pardo, 2006). However, it is easy to conceptualize neural activity as distinct from other forms of physiological evidence. For example, while we can manipulate neural activity by conducting mathematical problems in our head, we cannot change our DNA profile through thought processes. What legal weight such a distinction would carry is moot until tested in court. The more challenging question is whether or not authorities should be allowed to record our neural activity without our consent, or even our knowledge. When confronted with this problem courts will be forced to either shoehorn this new technology into existing legal frameworks governing conceptually similar subject matter (i.e., DNA, blood, fingerprints, etc.) or produce new bespoke legal frameworks for their governance. In the latter case, the form of any new framework cannot be predicted. A final difficult question is whether police could require a person to undergo an fMRI test without a warrant, with no clear consensus existing between commentators on this point (see Pardo, 2006 and Holley, 2009).
  Another set of issues is whether fMRI questioning undermines the right to silence and the right not to self-incriminate. Neuroimaging technology has the potential to undermine these rights if it can operate without the individual needing to speak. Within the United States, the Supreme Court has previously speculated that “the involuntary transmission of incriminating lie-detection evidence would violate a suspect's right to silence” (Simpson, 2008: 767). Under the European Convention on Human Rights (ECHR) whilst there is no explicit protection against self-incrimination, in the case of *Funke v France* (A/256-A, 1993; 1 C.M.L.R. 897, ECHR) the European Court of Human Rights (ECtHR) was explicit that the right not to self-incriminate is an implicit component of one's *Right to a fair trial* under Article 6 ECHR (Jackson, 2009) though it is not an absolute right (Berger, 2006). The ECtHR in *Saunders v United Kingdom* (1997, 23 EHRR 313) drew a distinction between material which respects the will of the suspect to remain silent and materials which exists independent of the suspect's will such as DNA, blood, urine, and breath. Unfortunately what they left for a future court to decide is whether or not an individual's brain activity exists independent of their will to remain silent?
  It must also be asked whether questioning in fMRI without consent engages Article 8 Right to respect for private and family life and Article 9 Freedom of thought, conscience and religion of the ECHR. Article 8(1) has been broadly interpreted in the past, and it is readily conceivable that processes which seek to determine the veracity of our statements by measuring neural activity will engage this right. The question will turn on whether or not police will be able to conduct questioning in fMRI under the Article 8(2) qualifications of national security, public safety, and crime prevention, and what protections will needed to ensure proportionality. The answer to this might well be tied with Article 9, for to allow the state to access thoughts without consent and knowledge may have a chilling effect on both individuals and society as they seek to exercise their freedom of thought. Courts may well-seek to impose stringent safeguards on neuroimaging technology to prevent both the overuse and misuse of them if they feel these rights are threatened.
  Additionally a number of rights within the Charter of Fundamental Rights of the European Union (CFREU) may also prove challenging for fMRI use in court proceedings. Article 1 *Human dignity* states that human dignity is inviolable and must be respected and protected. It is possible to argue that fMRI questioning without consent undermines an individual's dignity. Article 3 *Right to the integrity of the person* potentially poses the greatest challenge; especially Article 3(1) that everyone has the right to respect for their *physical* and *mental integrity*. This right may also be engaged by enforced or non-consensual fMRI questioning, especially given the express recognition of both physical and mental components. It is worth noting here that within France, Art.45 of LOI n°2011-814 of 7 July 2011 (i.e., post ratification of the CFREU) which created Art.16-14 of the French Civil Code^11^ specifically limits the use of brain imaging techniques to medical purposes, scientific research, and in the context of judicial enquiries carried out by experts. But most importantly that the express and informed consent of the individual *must be obtained in writing* prior to *any imaging*, and that this consent is revocable at any time. Given how few national legislators have specifically acknowledged the use of neuroimaging in judicial proceedings, let alone the issue of consent, this early approach by the French government takes on considerable significance.
  Finally Article 8 CFREU *Protection of personal data* poses a number of interesting questions. Firstly would fMRI data constitute personal data and thus fall under the protection of this article? Given the uniquely nature of such imaging in relation to an individual this must surely be the case. Assuming this is correct, under Article 8(2) everyone has the right to access their personal data and to have it rectified. As a result given the current fallibility of fMRI evidence, one could always argue that an interpretation of such data is incorrect and must be rectified. It would be interesting to see the effects on the admissibility of fMRI evidence in courts where one party seeks to challenge the ostensibly incorrect interpretation of their fMRI results by the other.
2. Compelled questioning and covert surveillance:
  The issues of compelled questioning and (as the technology develops) covert neuroimaging surveillance are ones which courts will be forced to face given the potentially profound impact covert surveillance of this nature will have on society as a whole. One of the concerns raised is the potential for authorities to use these technologies for *fishing trips* whereby police would question an individual to determine whether they have committed criminal acts in the past without any pre-existing evidence or reasonable suspicion. For police to search before they suspect is to undermine the presumption of innocence upon which our common law legal systems are built. Though as Pardo (2006) notes, it is not certain that such actions will be prevented under current regimes.
3. Probative value, unfair prejudice, and undermining the Province of the Jury:
  For evidence (including scientific evidence) to be admissible in criminal courts it must be *relevant*, thus possessing *probative value*. The probative value of evidence can be defined as “the extent to which [this evidence] increases or decreases the probability of a fact in issue” (Dennis, 2007, p.108). Thus, for fMRI evidence the probative value is the extent to which it increases or decreases the *subjective veracity probability* of a declarant's statement; i.e., how it affects the factual probability that a person does or does not believe what they are saying.
  However, probative value also refers to the *degree of relevance* evidence possesses, which is the extent to which evidence influences the probability of a fact in issue in the mind of a rational juror. Within England and Wales if the judge considers the probative value of evidence will have a prejudicial effect on this juror “disproportionate to the rational strength of the evidence as a means of proof, [then] the exclusionary discretion is available [to the judge] to prevent an accused suffering prejudice” (Dennis, 2007, p.108); thus the judge can exclude such disproportionate evidence. An example of evidence excluded may include full-color graphic photographs of injuries when a party seeks to admit these in addition to clear and factual medical reports. Similarly, within the US Federal Rules of Evidence (Rule 403, Federal Rules of Evidence) where the probative value of otherwise admissible evidence is substantially outweighed by the danger of unfair prejudice then this evidence too can be excluded. The question therefore becomes, will the courts reject *prima facie* admissible fMRI evidence on the basis that, because of its nature (or its presentation) it risks unfairly prejudicing the accused? Fears have been raised that: the graphic nature of fMRI evidence will result in unfair prejudice; that scientific lie detection evidence will unduly influence and taint jury deliberations; that jurors will not use their intuition and independent reasoning to critically challenge neuroimaging evidence; and that through function-creep such evidence will trespass into the *Province of the Jury* by effectively usurping their role as arbiter of fact (Gerard, 2008; see Weisberg et al., 2008 for evidence that fMRI images are seen as more compelling that other types and formats of data).
  Supporters of neuroscience technologies consider concerns over the undermining of judges and juries as unfounded; rather neuroimaging evidence will simply make the predictions of veracity by jurors and judges more reliable (Pardo, 2006). Pardo makes the argument that:
  Because even a highly reliable neuroscience test would not establish knowledge or lies directly, jurors would still need to play their traditional role in assessing it. In making these assessments, the jury would, for example, consider whether other evidence regarding credibility should override the test results, rendering the test conclusion unlikely. (2006: 318)
  While this statement seeks to defend and support fMRI in criminal courts, it unwittingly demonstrates the danger that this technology will import unfair prejudice into criminal trials. To explain; by rightly accepting that even a highly reliable neuroimaging test does not directly establish knowledge or lies one must ask “what is the point of introducing evidence to a jury from a technology that cannot provide direct evidence as to the veracity of statements made but is still marketed and promoted as a scientifically accurate lie detector^12^ ?” The obvious danger here is that the nuance between *lie detection* and *statement veracity* will not be clearly explained at the start of a case and/or not maintained and reinforced as the case progresses, leading juries to overestimate the capabilities of this technology. This is highlighted by the remainder of the above quote where the neuroimaging test results are already being presented by the author as the *de facto* position of truth, one which can only by overridden should *other evidence regarding credibility override the test results*; i.e., the tests shall be the truth unless you can prove otherwise. This statement, while seeking to defend neuroimaging technologies, actually serves to highlight the potential disproportional probative effect of neuroscience lie detectors. Cognitive neuroscientists must be careful not to overplay what these technologies can offer criminal courts nor their vision of the potential future role of neuroscience within criminal courts, lest they overplay themselves out of the courtroom altogether.
4. Right to a fair trial:
  Depending on how questioning in fMRI is conducted for criminal trials it can be argued that the fairness of trials will be placed at risk unless *all* parties to the trial are subjected to pre-trial questioning in fMRI. Presenting fMRI evidence from only one party to the case may result in an artificial disparity of evidence; i.e., the neuroimaging evidence plus testimony vs. testimony without neuroimaging evidence. Justice may now depend on whether or not a jury will question a technology promoted as a highly accurate lie detector, so to ensure parity of arms and a fair trial all parties should be subjected to pre-trial questioning in fMRI if they are ever introduced. Of course such a scenario depends on all the parties being capable of undergoing fMRI testing which is not the case when the victim is dead or comatose. In these circumstances fMRI evidence may need to be prohibited.
  Nevertheless, it is conceivable that fMRI evidence may become admissible solely as a defense instrument given that structural brain scans have already found acceptance and are widely admissible as mitigating evidence during sentencing proceedings. Indeed such use may help ensure a trial is ultimately fair. However, any attempt to extrapolate from this niche application such that fMRI evidence can be used throughout the entirety of the trial *but only by the defense* represents an arguably unacceptable asymmetry of measures; one which potentially undermines the overall fairness of the trial (both actual and perceived) and the rights of victims.

## Concluding points

Our discussion throughout has focused on the scientific, legal, and ethical hurdles facing those seeking to introduce fMRI evidence into trials as a means of assisting judges and juries in determining the veracity of statements made. Schauer (2010) suggests that as the goals of the law differ from those of science, what is not good enough for science may yet be good enough for the law and *vice versa*. However, following our assessments of the science underpinning fMRI as a lie detector and how this relates to the law, we must conclude that the current state of this technology, and potentially the technology *per se*, fails to meet either acceptable scientific or legal standards.

The evaluation of fMRI accuracy in lie detection—in some cases claimed to be as high as 0.90—is indeed based on laboratory experiments conducted with compliant participants, which is unlikely to be true of most legal settings where non-compliance and the use of countermeasures would make its accuracy figure drop dramatically (e.g., Ganis et al., 2011). In the cognitive neurosciences fMRI is not sufficient by itself to unveil which brain areas are epiphenomenal, which are strictly necessary to lying. It may thus pick up some noise together with the real signal. Even if it was possible to produce a correlational map where a constant pattern could be detected indicating a lie, issues of replicability and generalizability across conditions and participants could be raised. And even more so in those cases where facts are unknown to the tester and there is no objective reality against which to establish whether a person is lying or not. From the legal perspective, until the science behind fMRI testing improves it will not meet the relevance and reliability thresholds required for any scientific evidence to be admissible in criminal trials. The assumptions, inferences, and questions of internal validity which so pervade current fMRI testing and analysis need to be addressed. As does the challenge of successfully applying this technology to criminal justice scenarios characterized by their confrontational emotional nature and the personal high-stakes involved for the participants.

Neuroimaging in courts also raises the specter of potential constitutional and human rights violations. Questions arise as to whether or not such testing constitutes an illegal search as well as how it respects rights to privacy, silence, thought, and a fair trial are all engaged by this technology yet left unanswered. Unless the admissibility decision is taken out of the hands of the judiciary by politicians, which is itself a likely scenario, ultimately it will be for the courts to decide the fate of fMRI evidence in criminal trials. Given the range and depth of the legal and ethical issues identified in the earlier sections, the likely outcome will probably fall on a spectrum somewhere between outright rejection through to some form of restricted and regulated usage, as opposed to the highly unlikely scenario of *carte blanche* acceptance.

What we have not discussed within this paper are both the *operational* and *social* barriers to the widespread use of fMRI testing in criminal trials. These barriers are potentially just as daunting as their scientific and legal counterparts.

From the *practical operational perspective*, issues which require future examination include; the cost of purchasing, staffing, and maintaining sufficient fMRI machines to cater for a national justice system; the additional time and monetary costs fMRI testing will add to criminal cases; how fMRI testing can be made to work within adversarial systems of questioning and cross-examination based on earlier responses; and the lack of a courtroom-friendly portable fMRI system. Additionally there are questions specific to the assessment algorithms used when interpreting fMRI response data: will only a single universal *official* algorithm be allowed?; will commercial patented algorithms be admissible if they are not completely open for inspection and independent verification?; and finally what happens when new algorithms and new fMRI scanners are inevitably developed as the science is continually refined which prove to be more reliable and sensitive than previous algorithms/machines? It is conceivable that those who maintain their innocence and are appealing their conviction under the previous technology will seek to be re-tested with the new machines and the new algorithm in an effort to prove their innocence placing a further burden on the criminal justice system. All of these points possess the potential to impact upon the fairness of future trials.

A final hurdle to the widespread introduction of fMRI testing is *societal acceptability*, without which technologies such as neuroimaging techniques for determining the veracity of statements within criminal trials will lack both public confidence and legitimacy. Future research needs to gauge the levels of public support for such technologies, for even if neuroimaging proves superior to humans as arbiters of statement veracity in criminal courts, this fact in of itself may not be enough for the public to accept their introduction if they are apprehensive or hostile to what such technologies represent for their future. We cannot escape from asking the question, *will people accept mind reading machines?* This is obviously not what the current generation of neuroimaging technologies is, but they are a small step down this long path.

Our societies have developed to both accept and respect an individual's right to keep secrets, and in so doing they do not seek to override human beings' evolved capacity to keep secrets, for a society where individuals are denied secrets is not a human society as we know it. The developers and proponents of fMRI testing must respect this fact and engage society in their research as it progresses. Otherwise they may find they successfully negotiate the frying-pan of scientific and technical challenges in perfecting fMRI testing only to be consumed by a fire of legal, ethical, social, and political opposition.

### Conflict of interest statement

The authors declare that the research was conducted in the absence of any commercial or financial relationships that could be construed as a potential conflict of interest.

## Footnotes

^1^A pulse sequence is the series of changing magnetic field gradients and oscillating electromagnetic fields defined by the user that allows the MRI scanner to tune on and create images sensitive to a target physical property. Different pulse sequences, for example, are used when collecting structural data and functional brain activations.

^2^The term “functional” refers to changes in brain function and regional levels of activation over time.

^3^fMRI is based on the difference in magnetic resonance signals from oxyhemoglobin and deoxyhemoglobin and builds on the fact that active brain regions tend to use more oxygen than relatively inactive regions. Soon after a brain region has been activated by a cognitive event or task, the local microvasculature responds to increased oxygen consumption by increasing the flow of new arterial blood (i.e., blood rich in oxyhemoglobin) to the region. As a consequence, the relative concentration of deoxyhemoglobin decreases, thus causing localized changes in the magnetic resonance signal. These changes are known as blood oxygenation level-dependent (BOLD) signal (Purves et al., 2013).

^4^For a broad overview on other relevant testing paradigms—that can also be used in fMRI studies—we direct the reader toward Gamer (2011). For an insightful discussion on the complexity of deception and the unlikelihood of encompassing it by using simple tasks that require participants to lie in response to certain stimuli and tell the truth in response to others, we direct the reader toward Sip et al. (2007). Our discussion will provide prototypical examples in order to highlight general principles; it is by no means aimed to provide a comprehensive and systematic description of the vast literature on the topic.

^5^Internal Validity refers to the appropriateness of construct operationalization and of experimental design in order to test the hypothesis of interest. It guarantees that any obtained effects may be univocally attributed to the experimental manipulation. Clearly, the use of expensive and fancy techniques does not guarantee by itself that experimental results are meaningful and interpretable.

^6^In this context, the “construct of interest” is the brain fingerprint of lying.

^7^The difference in signal on fMRI images from different experimental conditions as a function of the amount of deoxygenated hemoglobin.

^8^The term “parieto-frontal” areas denotes brain regions in the parietal and frontal lobes. The parietal lobe is located on the posterior and dorsal surfaces of the cerebrum. The frontal lobe is the most anterior lobe of the cerebrum.

^9^In some states polygraph evidence is permitted when both the prosecution and defense agree to its admissibility, while in others such evidence cannot be admitted even when both parties would otherwise agree.

^10^The sources examined here are: the US Constitution (limited in application to US citizens within US territories), the European Convention on Human Rights (produced by, and applying to, the 47 member states of the Council of Europe, and overseen by the European Court of Human Rights), and the Charter of Fundamental Rights of the European Union (applicable to all citizens and residents of the 28 member states of the EU, this Charter enshrines a range of personal, civil, and social rights and existing conventions and treaties (including the European Convention on Human Rights) into EU law thus ensuring their legal certainty).

^11^Taken from the French Civil Code, Book I: People, Title I: The Civil Rights, Chapter IV: the use of brain imaging techniques.

^12^Both of the two commercial companies offering fMRI detection service specifically and deliberately promote their technologies as scientific lie detection tools and not as veracity probability enhancement tools; No Lie MRI claims their technology ‘represents the first and only direct measure of truth verification and lie detection in human history’ (see http://noliemri.com/), while CEPHOS Corp claims to have developed ‘the latest, most scientifically advanced, brain imaging techniques for scientifically accurate lie detection’ (see http://www.cephoscorp.com/).
